# Safety and effectiveness of enhanced external counterpulsation (EECP) in refractory angina patients: A systematic reviews and meta-analysis

**DOI:** 10.34172/jcvtr.2021.50

**Published:** 2021-11-23

**Authors:** Seyed Mansoor Rayegani, Saeed Heidari, Majid Maleki, Maryam Seyed-Nezhad, Maryam Heidari, Seyed Ehsan Parhizgar, Mohammad Moradi-Joo

**Affiliations:** ^1^Physical Medicine and Rehabilitation Research Center, Shahid Beheshti University of Medical Sciences, Tehran, Iran; ^2^State Welfare Organization of Iran, Director General of Welfare of Kermanshah Province, Kermanshah, Iran; ^3^Cardiogenetic Research Center, Rajaie Cardiovascular Medical and Research Center, Iran University of Medical Sciences, Tehran, Iran; ^4^Department of Health Management and Economics, School of Public Health, Tehran University of Medical Sciences, Tehran, Iran; ^5^Shahid Beheshti Hospital, Kashan University of Medical Sciences, Kashan, Iran; ^6^National Center for Health Insurance Research, Tehran, Iran

**Keywords:** Safety, Effectiveness, EECP, Angina, Systematic Reviews, Meta-Analysis

## Abstract

Enhanced external counterpulsation (EECP) is believed to be a non-invasive treatment for coronary artery disease and angina. The aim of this study was to determine the safety and effectiveness of EECP in refractory angina patients through a systematic reviews and meta-analysis. We conducted a comprehensive search of the literature published on PubMed, Cochrane library, Scopus, ScienceDirect, Trip Database and Google Scholar databases using appropriate keywords and specific strategy with no time limit. Having selected and screened the studies based on the defined inclusion and exclusion criteria and evaluating their quality based on the Cochrane checklist. For the meta-analysis,the Mantel-Haenszel method or the generic Inverse Variance was used. Analyses were done with Review Manager 5.2 software. A number of 299 studies were initially reviewed and finally, seventeen studies were included in the meta-analysis based on the inclusion and exclusion criteria. Also, thirteen outcomes were analyzed and the results of meta-analysis in twelve outcomes including (Systolic Blood Pressure (7 studies), Diastolic Blood Pressure (7 studies), Pulse Pressure (4 studies), Mean Arterial Pressures (4 studies), Heart Rate (6 studies), Angina episodes (7 studies), Walking distance (2 studies),Canadian Cardiovascular Society classification (6 studies), Flow-Mediated Dilation (3 studies), Daily Nitrate Usage (4 studies), Exercise Treadmill Test-Time (2 studies), ST-segment depression (2 studies)demonstrated a significant clinical advantage in the EECP treatment effectiveness in patients with angina. No significant difference was observed regarding EECP usefulness (*P* = 0.18) in the outcome of brachial artery diameter (2 studies). Based on the meta-analysis, the results indicate the safety and effectiveness of EECP in patients with angina pectoris and indicate the usefulness of this treatment in these patients. In general, the authors believe that the general conclusion in this regard requires some studies with a large sample size and a control group assignment.

## Introduction

The cardiovascular disease epidemic has plagued most countries worldwide. Although many cardiovascular diseases can be treated, it is still the leading cause of death in men and women throughout the world.^1^

According to the World Health Organization (WHO), 17.9 million people died of cardiovascular disease in 2019, accounting for 32% of all deaths per year. Of course, 85% of deaths are due to heart attack and stroke.^2^ Cardiovascular mortality is predicted to account for more than 23.6 million per year by 2030.^3^ The total cost incurred due to cardiovascular disease is reported to be $ 177.5 billion annually.^4^

Cardiovascular diseases can be prevented through addressing behavioral risk factors such as smoking, unhealthy diet, obesity, physical inactivity and persistent alcohol use. People with cardiovascular disease or people at high cardiovascular risk need to be diagnosed and managed early through counseling and medication, if required.^2^ Although drug and invasive treatments have increased life expectancy among many patients, many of these patients are resistant to these treatments.Angina, also known as angina pectoris, is chest pain, usually caused by blockage or spasm of the arteries that carry blood to the heart muscle.^5^

Some patients with angina neither adequately react to medication, nor they respond well to myocardial revascularization. However, the desired treatment outcome cannot be achieved in some of these patients, because the risk-benefit ratio is not very considerable for myocardial revascularization. However, in some of these patients, the desired treatment outcome cannot be achieved because the risk-benefit ratio is not very attractive for myocardial revascularization. In all of these cases, enhanced external counterpulsation (EECP) can be used as a potential treatment for chronic refractory angina.^6^

EECP approved by the United States Food and Drug Administration in 1995. EECP was first introduced in the treatment of angina and was subsequently applied in various conditions including heart failure, ischemic cerebrovascular diseases and cardiomyopathy. EECP is a non-invasive mechanical, outpatient treatment for coronary artery disease (CAD) patients with refractory angina pectoris. Various studies have shown that EECP improves the symptoms of angina, myocardial ischemia, left ventricular function, and quality of life.^7-10^ Patients for whom EECP treatment is effective may experience lasting benefits for up to 5 years after treatment. Therefore, EECP may be a long-term, cost-effective, non-invasive treatment for chronic angina.^11,12^

Owing to the significant prevalence of refractory angina syndrome in population groups and the extent of results regarding the safety and effectiveness of EECP in the treatment of patients suffering from chronic angina, the present study was conducted as a systematic review and meta-analysis. The aim of this study was to determine the safety and effectiveness of EECP in refractory angina patients.

## Methods

### Search Strategy

Keywords including (EECP, enhanced external counterpulsation, external counter pulsation, angina) in PubMed, Cochrane library, Scopus, Science Direct, Trip databases were applied to garner research studies that reported on the safety and effectiveness of EECP technology with no time limit until May 2021.

For each database, a specific and appropriate search strategy was used according to query and its structured components. The Google Scholar search engine was used to look up relevant resources and complete the search coverage. The searched studies references were also used to find the related studies.

### Selection of Studies

Initially, the studies obtained from electronic search and manual search were organized using EndNote software followed with screening and selection of studies through two stages based on inclusion and exclusion criteria. In the first stage, the titles and abstracts of the studies were reviewed after omitting the duplicate studies, and, the full text of the selected studies was collected and reviewed in the second stage. Then, the list of sources of the remaining studies was reviewed and, if necessary, the corresponding authors of important studies were contacted. Screening and selection of studies were performed by two reviewers (SH and MMJ) independently.

Finally, the process of searching and selecting studies was mapped using the PRISMA flow chart.

### Eligibility criteria

**Population:** Angina patients;

**Intervention:** Enhanced external counterpulsation (EECP);

**Outcome:** Clinical efficacy, functional consequences, side effects and safety;

**Type of studies:** Trial and observational studies

Exclusion criteria were that; 1) Studies published in languages other than English or Persian; 2) Studies whose statistical data were incomplete or not reported at all; 3) Studies that have not evaluated the intended outcomes; 4) Studies without explicit methodology or results; 5) Studies whose population was arrhythmias leading to dysfunction, active thrombophlebitis, aortic aneurysms, significant valvular diseases, or pregnant women.

### Risk of bias/quality assessment

Cochrane checklist were used to assess the risk of bias tool that assesses the risks of selection, performance, detection, attrition, and reporting.^13^ Two reviewers independently assessed the risk of bias, and disagreements were resolved through discussion or consulting the third reviewer.

### Data extraction

Two independent reviewers extracted the data. In addition, we extracted the following variables: title of article, first author, year of publication, country, number of patient, type of study, statistical data related to each outcome, outcome follow-up time, and other useful information. After filling out the data extraction forms (designed in Excel), the disagreements were resolved through discussion or consulting the third reviewer.

### Statistical analysis

Mean Difference (MD) and Inverse Variance (IV) method were used to pool the data in cases where the data was binary, the relative risk was used to pool the data using the Cochran–Mantel–Haenszel test (CMH) test. Pooled estimates were performed using a random-effects model in case of heterogeneity; otherwise, fixed-effects model was used. Statistical heterogeneity was evaluated using means of chi-square and I^2^ statistics. The I^2^ more than or equal to 40% was considered high statistical heterogeneity.^14^ The proportions and 95% confidence intervals (CIs) for each categorical factor. The meta-analysis was performed using RevMan Software (version 5.2).

## Results

### Study selection and characteristics

Databases search revealed 272 studies in the first stage, manual search and review added 27 studies to this number, which totaled 299 studies. Having deleted the common titles, we carried out an initial screening of 127 studies. After studying the title and abstract, 88 studies were omitted due to inconsistency with the purpose of the study. The full text of 39 studies was then assessed for eligibility. Finally, 17 studies were included in quantitative synthesis.

**Figure 1:**
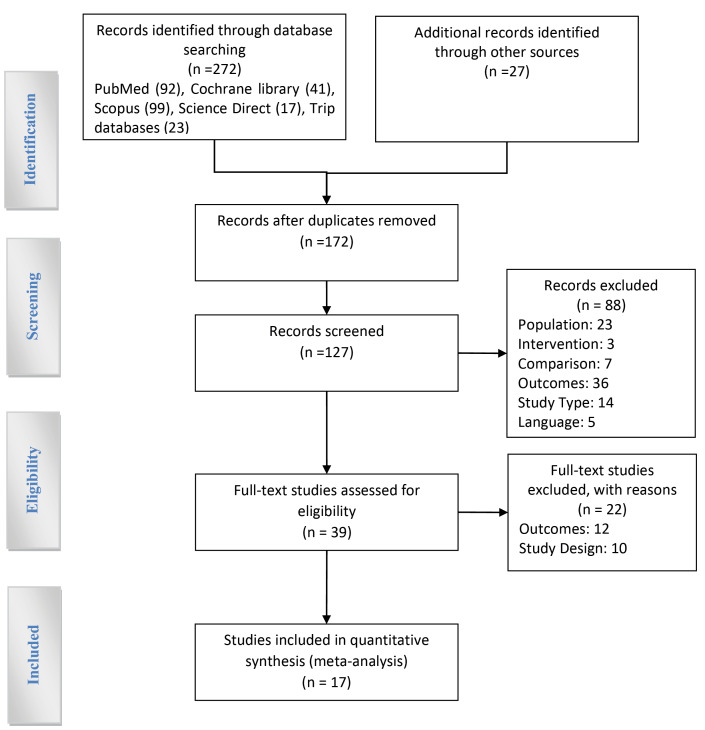
Flow diagram for selection of studies for the systematic review and meta-analysis (PRISMA Diagram)

Figure 1 shows the process of searching and applying the inclusion and exclusion criteria of studies.

All the seventeen studies reviewed the desired outcomes and related statistical data before and after treatment of refractory angina pectoris patients with EECP. Of these seventeen studies, sixteen were clinical trials in which the total study population are 582 patients and was a type of cohort in which the study population included 450 patients. The mean age of patients was 27.1 years in the Gurovich study15 and 76.8 years in the Braverman study16 All studies were published between 1999 and 2020, (Table 1).

**Table 1:** Main characteristics of included studies

**First author**	**Year**	**Country**	**Study Design**	**Mean age±SD**	**Sample Size**	**Population**	**Follow-up**	**EECP** ^*^ **Sessions**
Arora^17^	1999	USA (New York)	Clinical trials	64 ± 9	71	Angina and coronary artery disease	4-7 weeks	35 1-hour sessions
Beck^18^	2014	USA (Rhode Island)	Clinical trials	64 ± 8	25	Left ventricular dysfunction & coronary artery disease	7 weeks	35 1-hour sessions
Beck^19^	2015	USA (Rhode Island)	Clinical trials	64.2 ± 2.6	10	Left ventricular dysfunction	7 weeks	35 1-hour sessions
Bondesson^20^	2010	Sweden	Clinical trials	69	100	Refractory angina pectoris	12 months	35 1to 2-hour sessions
Braith^21^	2010	USA (Florida)	Clinical trials	64.44 ± 9.63	28	Coronary artery disease	7 weeks	35 1- hour sessions
Braverman^16^	2013	USA (Pennsylvania)	Clinical trials	76.8 ± 7	86	With Aortic Stenosis	6 weeks	N.R
Casey^22^	2008	USA (Rochester)	Clinical trials	63 ± 11	12	Angina pectoris	7 weeks	35 1-hour sessions
Casey^23^	2011	USA (Rochester)	Clinical trials	64 ± 2	28	Chronic Angina Pectoris	7 weeks	35 1- hour sessions
Dockery^24^	2004	UK	Clinical trials	63.7 ± 6.7	23	Patients with Angina	7 weeks	35 1-hour sessions
Gurovich^15^	2013	USA	Clinical trials	27.1 ± 5	18	Coronary artery disease and unstable angina	4-7 weeks	35 45-min to 1-hour sessions
Hashemi^25^	2008	Iran	Clinical trials	63.93 ± 8.6	15	Ischemic cardiomyopathy	1 month	35 1-hour sessions
Kumar^26^	2009	USA (New York)	Clinical trials	61 ± 8	47	Prior coronary revascularization who had chronic refractory angina pectoris	12 months	35 1-hour sessions
Michaels^27^	2007	USA	Clinical trials	62 ± 10	24	Chronic stable angina due to coronary artery disease	7 weeks	35 1-hour sessions
Nichols^28^	2006	USA (Florida)	Clinical trials	61 ± 7.1	20	Refractory angina pectoris	7 to 8 weeks	35 1-hour sessions
Soran^29^	2007	USA	Cohort	69 ± 11	450	Refractory angina and left ventricular (LV) dysfunction	6 months	35 1-hour sessions
Tartaglia^30^	2003	USA (New York)	Clinical trials	68 ± 9	25	Angiographically proven coronary artery disease	4-7 weeks	35 1-hour sessions
Wu^31^	2020	Sweden	Clinical trials	65.8	50	Refractory Angina Pectoris	6 months	35 1-hour sessions

^*^Abbreviations: EECP, enhanced external counterpulsation; N.R, No Report

### Risk of bias in included studies

**Figure 2:**
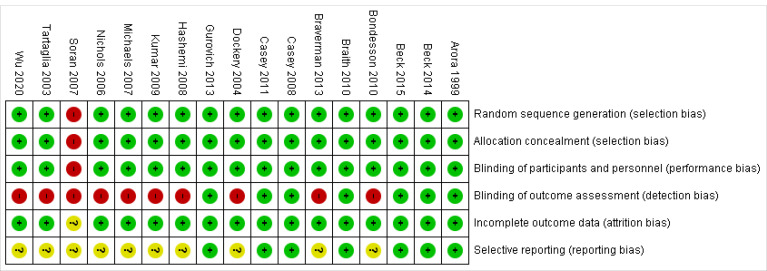
Risk of bias item for each included study low risk bias, high-risk bias, unclear risk

Risk of bias assessment of the studies showed that, 7 studies out of 17 studies were of good quality (low risk bias), one study was of low quality (high risk bias) and 9 studies were of moderate quality. The most important challenge for studies in Cochrane risk of bias items was related to item blinding of outcome assessment (detection bias). Also in the item selection reporting (reporting bias) 59% of the studies were unclear risk (Figure 2).

### Outcomes

After categorizing the outcomes of the 17 studies included in the present study, 13 outcomes were extracted: Systolic Blood Pressure (7 studies), Diastolic Blood Pressure (7 studies), Pulse Pressure (4 studies), Mean Arterial Pressures (4 studies), Heart Rate (6 studies), Angina episodes (7 studies), Walking distance (2 studies), Canadian Cardiovascular Society (6 studies), Flow-Mediated Dilation (3 studies), Daily Nitrate Usage (4 studies), Exercise Treadmill Test-Time (2 studies), ST-segment depression (2 studies) and brachial artery diameter (2 studies).11 outcomes were analyzed for clinical efficacy and functional consequences (Effectiveness) and 2 outcomes were analyzed as safety criteria.

### Effectiveness

#### Systolic Blood Pressure (SBP)

**Figure 3:**
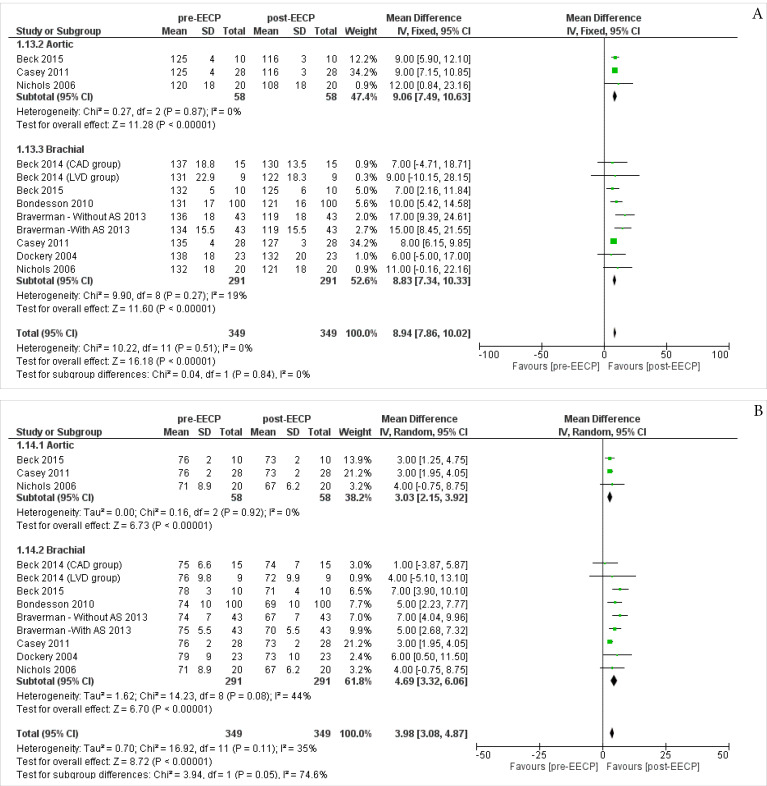
Forest plot analysis of the (A) SBP (B) DBP

Three studies^19,23,28^ including 58 patients assessed the Aortic SBP outcomes of the patients. The overall Inverse Variance Pooled MD calculated for Aortic SBP was 9.06 (95% CI; 7.49, 10.63) in favor of the post-EECP (*P* < 0.00001, Figure 3A). Test for heterogeneity was not statistically significant (I^2^ = 0%, *P* = 0.87).

Seven studies^16,18-20,23,24,28^ including 291 patients assessed the Barchial Systolic Blood Pressureoutcomes of the patients. The overall Inverse Variance Pooled MD calculated for Barchial SBP was 8.83 (95% CI; 7.34, 10.33) in favor of the post-EECP (*P* < 0.00001, Figure 3A). Test for heterogeneity was not statistically significant (I^2^ = 19%, *P* = 0.27).

The overall Inverse Variance Pooled MD calculated for Aortic and Barchial SBP was 8.94 (95% CI; 7.86, 10.02) in favor of the post-EECP (*P* < 0.00001). Test for heterogeneity was not statistically significant (I^2^ = 0%, *P* = 0.51).

#### Diastolic Blood Pressure (DBP)

Three studies^19,23,28^ including 58 patients assessed the Aortic DBPoutcomes of the patients. The overall Inverse Variance Pooled MD calculated for Aortic DBP was 3.03 (95% CI; 2.15, 3.92) in favor of the post-EECP (*P* < 0.00001, Figure 3B). Test for heterogeneity was not statistically significant (I^2^ = 0%, *P* = 0.92).

Seven studies^16,18-20,23,24,28^ including 291 patients assessed the Barchial DBPoutcomes of the patients. The overall Inverse Variance Pooled MD calculated for Barchial DBP was 4.69 (95% CI; 3.32, 6.06) in favor of the post-EECP (*P* < 0.00001, *P*4). Test for heterogeneity was not statistically significant (I^2^ = 44%, *P* = 0.08).

The overall Inverse Variance Pooled MD calculated for Aortic and Barchial DBP was 3.98 (95% CI; 3.08, 4.87) in favor of the post-EECP (*P* < 0.00001). Test for heterogeneity was not statistically significant (I^2^ = 35%, *P* = 0.11).

#### Pulse Pressure (PP)

**Figure 4:**
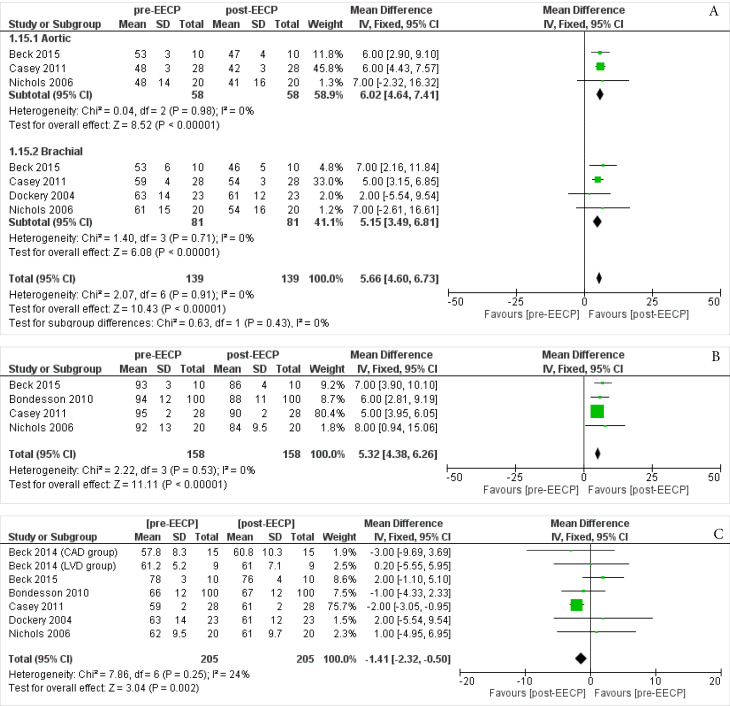
Forest plot analysis of the (A) PP (B) MAP (C) Heart Rate

Three studies^19,23,28^ including 58 patients assessed the Aortic PPoutcomes of the patients. The overall Inverse Variance Pooled MD calculated for Aortic PP was 6.02 (95% CI; 4.64, 7.41) in favor of the post-EECP (*P* < 0.00001, Figure 4A). Test for heterogeneity was not statistically significant (I^2^ = 0%, P = 0.98).

Four studies^19,23,24,28^ including 81 patients assessed the Barchial PPoutcomes of the patients. The overall Inverse Variance Pooled MD calculated for Barchial PP was 5.15 (95% CI; 3.49, 6.81) in favor of the post-EECP (*P* < 0.00001, Figure 4A). Test for heterogeneity was not statistically significant (I^2^ = 0%, *P* = 0.71).

The overall Inverse Variance Pooled MD calculated for Aortic and Barchial PP was 5.66 (95% CI; 4.60, 6.73) in favor of the post-EECP *(P* < 0.00001). Test for heterogeneity was not statistically significant (I^2^ = 0%, *P* = 0.91).

#### Mean Arterial Pressures(MAP)

Four studies^19,20,23,28^ including 158 patients assessed the MAP outcomes of the patients. The overall Inverse Variance Pooled MD calculated for MAP was 5.32 (95% CI; 4.38, 6.26) in favor of the post-EECP (*P* < 0.00001, Figure 4B). Test for heterogeneity was not statistically significant (I^2^ = 0%, *P* = 0.53).

### Heart Rate

Six studies^18-20,23,24,28^ including 205 patients assessed the Heart Rate outcomes of the patients. The overall Inverse Variance Pooled MD calculated for Heart Rate was -1.41 (95% CI; -2.32, -0.50) in favor of the post-EECP (*P* = 0.002, Figure 4C). Test for heterogeneity was not statistically significant (I^2^ = 24%, *P* = 0.25).

### Walking distance

**Figure 5:**
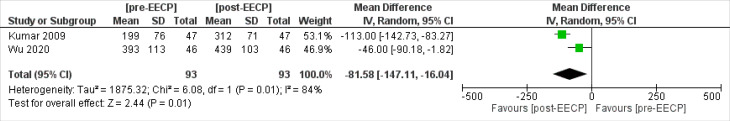
Forest plot analysis of the walking distance

Two studies^26,31^ including 93 patients assessed the walking distance outcomes of the patients. The overall Inverse Variance Pooled MD calculated for walking distance was -81.58 (95% CI; -147.11, -16.04) in favor of the post-EECP (*P* = 0.01, Figure 5). Test for heterogeneity was statistically significant (I^2^ = 84%, *P*= 0.01).

### Brachial Artery Diameter

**Figure 6:**
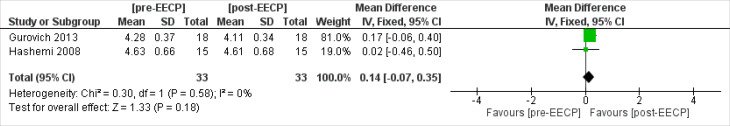
Forest plot analysis of the Brachial Artery Diameter

Two studies^15,25^ including 33 patients assessed the brachial artery diameter outcomes of the patients. The overall Inverse Variance Pooled MD calculated for brachial artery diameter was 0.14 (95% CI; -0.07, 0.35). The result of meta- analysis this outcome showed that there was no statistically significant difference between post-EECP & pre-EECP in terms of this outcome (*P* = 0.18, Figure 6). Test for heterogeneity was statistically significant (I^2^ = 84%, *P* = 0.01).

### Flow-Mediated Dilation(FMD)

**Figure 7:**
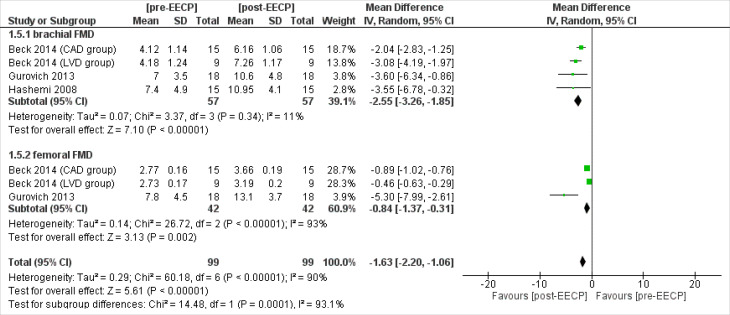
Forest plot analysis of the Flow-Mediated Dilation (FMD)

Three studies^15,18,25^ including 57 patients assessed the brachial FMD outcomes of the patients. The overall Inverse Variance Pooled MD calculated for brachial FMD was -2.55 (95% CI; -3.26, -1.85) in favor of the post-EECP *(P* < 0.00001, Figure 7). Test for heterogeneity was not statistically significant (I^2^ = 11%,* P* = 0.34).

Two studies^15,18^ including 42 patients assessed the femoral FMD outcomes of the patients. The overall Inverse Variance Pooled MD calculated for femoral FMD was -0.84 (95% CI; -1.37, -0.31) in favor of the post-EECP (*P* = 0.002, Figure 7). Test for heterogeneity was statistically significant (I^2^ = 93%, *P* < 0.00001).

### Exercise Treadmill Test-Time

**Figure 8:**
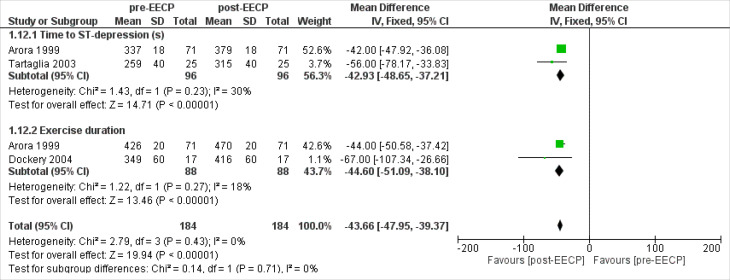
Forest plot analysis of the Exercise Treadmill Test-Time

Two studies^17,30^ including 96 patients assessed the time to ST depression outcomes of the patients. The overall Inverse Variance Pooled MD calculated for time to ST depression was -42.93 (95% CI; -48.65, -37.21) in favor of the post-EECP (*P* < 0.00001, Figure 8). Test for heterogeneity was not statistically significant (I^2^ = 30%, *P* = 0.23).

Two studies^17,24^ including 88 patients assessed the exercise duration outcomes of the patients. The overall Inverse Variance Pooled MD calculated for exercise duration was -44.60 (95% CI; -51.09, -38.10) in favor of the post-EECP (*P* < 0.00001, Figure 8). Test for heterogeneity was not statistically significant (I^2^ = 18%, *P* = 0.27).

### ST-segment depression

**Figure 9:**
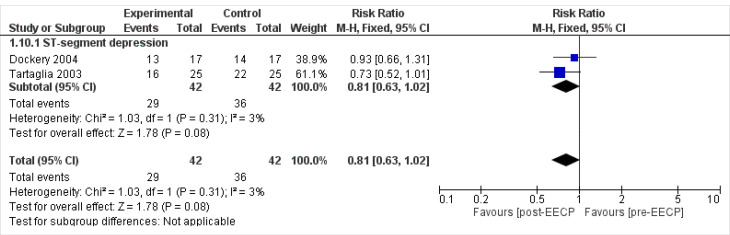
Forest plot analysis of the ST-segment depression

Two studies^24,30^ including 42 patients assessed the ST depression outcomes of the patients. The overall Mantel-Haenszel Pooled RR calculated for ST depression was 0.81 (95% CI; 0.63, 1.02). The result of meta- analysis this outcome showed that there was no statistically significant difference between post-EECP & pre-EECP in terms of this outcome (*P* = 0.08, Figure 9). Test for heterogeneity was not statistically significant (I^2^ = 3%, *P* = 0.31).

### CCS classification (CCS angina class)

**Figure 10:**
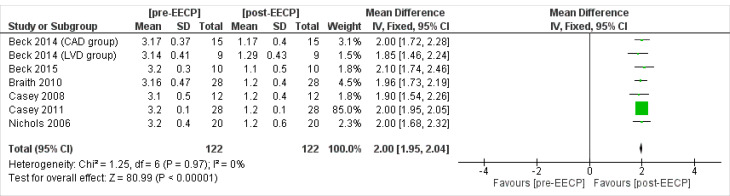
Forest plot analysis of the CCS angina class

Six studies^18,19,21-23,28^ including 122 patients assessed the Canadian Cardiovascular Society (CCS) angina class outcomes of the patients. The overall Inverse Variance Pooled MD calculated for CCS angina class was 2 (95% CI; 1.95, 2.04) in favor of the post-EECP (*P* < 0.00001, Figure 10). Test for heterogeneity was not statistically significant (I^2^ = 0%, *P* = 0.97).

### Safety

#### Angina episodes

**Figure 11:**
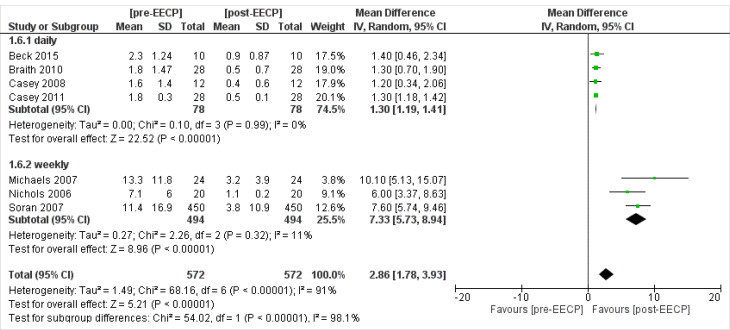
Forest plot analysis of the Angina episodes

Four studies^19,21-23^ including 78 patients assessed the daily angina episodes outcomes of the patients. The overall Inverse Variance Pooled MD calculated for daily angina episodes was 1.30 (95% CI; 1.19, 1.41) in favor of the post-EECP (*P* < 0.00001, Figure 11). Test for heterogeneity was not statistically significant (I^2^ = 0%, *P* = 0.99).

Three studies^27-29^ including 494 patients assessed the weekly angina episodes outcomes of the patients. The overall Inverse Variance Pooled MD calculated for weekly angina episodes was 7.33 (95% CI; 5.73, 8.94) in favor of the post-EECP (*P* < 0.00001, Figure 11). Test for heterogeneity was not statistically significant (I^2^ = 11%, *P* = 0.32).

### Daily Nitrate Usage

**Figure 12:**
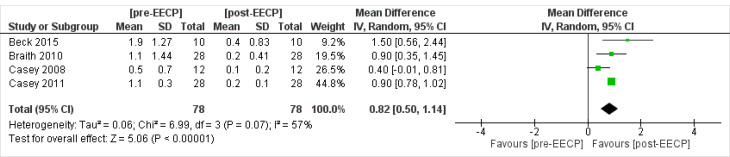
Forest plot analysis of the Daily Nitrate Usage

Four studies^19,21-23^ including 78 patients assessed the daily nitrate usage outcomes of the patients. The overall Inverse Variance Pooled MD calculated for daily nitrate usage was 0.82 (95% CI; 0.50, 1.41) in favor of the post-EECP (*P* < 0.00001, Figure 12). Test for heterogeneity was not statistically significant (I^2^ = 57%, *P* = 0.07).

## Discussion

A total of 299 studies were obtained through systematic search based on the predefined inclusion and exclusion criteria, and finally seventeen studies would effectively pertain to the desired consequences before and after treatment of patients with angina. Of these 17 studies, 16 are clinical trials with a total population of 582 patients, only one cohort study with a population of 450 patients. The mean age of patients was 27.1 years in the Gurovich study^15^ and 76.8 years in the Braverman study.^16^ All studies were published between 1999 and 2020. The evaluation of these studies quality, showed that, sixteen studies out of 17 studies submitted were of high quality and only one article was of low quality.

In this study, thirteen outcomes were analyzed and the results of meta-analysis in twelve outcomes indicate a significant clinical advantage in terms of the usefulness of EECP treatment in patients with angina. The effects of Aortic and Barchial SBP and Aortic and Barchial DBP in seven studies and the consequences of Aortic and Barchial PP and MAP in four studies were significantly reduced after treatment with EECP (*P* < 0.00001). The outcomes of Heart Rate, walking distance, and Exercise Treadmill Test-Time which significantly increased after treatment with EECP were reported in six, two and three studies, respectively (*P* < 0.00001). The outcome of angina episodes which was evaluated in four studies on daily basis and three studies weekly and also the outcome of CCS angina which was evaluated from a meta-analysis of six studies and the outcome of daily nitrate consumption which was evaluated from a meta-analysis of four studies revealed significantly reduced outcomes after treatment with EECP (*P* < 0.00001). The outcome of brachial and femoral FMD, which was examined in three studies, was significantly increased after treatment with EECP (*P* < 0.00001).No significant difference was observed in terms of usefulness in treatment with EECP (*P* = 0.18) only in the outcome of brachial artery diameter, which was the result of meta-analysis of two studies. The effect size index was the mean difference in all outcomes, except one outcome (t-segment depression) which used relative risk, whose results also indicate a significant outcome in favor of EECP treatment.

The outcomes meta-analysis results showed that, the I^2^ statistic, which is the criterion for the presence of heterogeneity, was calculated below 25% except for the outcomes of walking distance and femoral flow-mediated dilation, which indicates the absence of significant heterogeneity. This in turn, increases the reliability and general and definite conclusions about the effectiveness and safety of this treatment and its generalizability.

In a systematic review and meta-analysis conducted by Zhang et al^32^ in 2015, only were the implications of the CCS Angina Class examined. The present study in which thirteen outcomes have been evaluated and meta-analyzed is comprehensive and up-to-date. Amin et al^33^ systematic review in 2010 examined the effectiveness of EECP in young 18-year-olds, in which only one randomized controlled trial (RCT) article which compared EECP with sham was evaluated, whereas the present study is 10 years newer and more up-to-date than the study in question. In addition, studies that examined the EECP with the placebo group or other treatments such as medication were limited, hence in this study the effectiveness of EECP was evaluated before and after treatment. Of course, evaluating the effectiveness and safety of the EECP before and after treatment faces the bias, which reduces the validity and generalizability of the results thanks to the bias associated with the study design of such RCTs. Another systematic review and meta-analysis was performed to assess whether EECP affects myocardial perfusion in CAD patients. It has been shown that standard EECP treatment significantly increases myocardial perfusion in CAD patients.^5^

One of the limitations of this study was the short follow-up time of the results in the studies submitted for analysis. To the best of our knowledge and based on the studies submitted, there was no evidence of examining the outcomes of long-term follow-up periods (more than one year), and there is a large study gap in this regard, accordingly. It seems that we need to conduct studies with a follow-up time of more than one year in order to make a more realistic decision and judgment about the therapeutic role of EECP. Other limitations of this study are the small sample size of each study and the consequent low sample size of the meta-analyzed consequences. Also, articles whose statistical data were not fully reported were not included in the study. Therefore, two articles were not included in the study because their statistical data were not fully reported and we did not have access to their statistical data.^34,35^ We tried to contact the author of these studies, but unfortunately, we were not able to receive any related data, and the incompleteness of the statistical data may affect the results.

## Conclusion

Although, the results significantly indicate the safety and effectiveness of EECP in patients with angina pectoris and indicate the usefulness of this treatment in these patients based on the meta-analyzes; in general, the authors believe that, the general conclusion in this case requires high quality studies along with larger sample size and a control group due to the afro-mentioned limitations and the existence of evidence with low sample size as well as the examination of the outcomes before and after the treatment, though.

## Acknowledgments

The authors would like to thank the following individuals who have contributed at various stages through the development of this project

## Competing interest

The authors declare that there are no conflicts of interest.

## Ethical approval

The study was approved by the local ethical committee (code: IR.SBMU.RETECH.REC.1398.829) and the Helsinki Declaration was respected across the study.

## Funding/Support

The project has been supported by of Shahid Beheshti University of Medical* Sciences*
